# THE IMPACT OF RACE AND GENDER ON OVERALL CARE RATING AT THE END OF LIFE: APPLYING THE THEORY OF INTERSECTIONALITY

**DOI:** 10.1093/geroni/igac059.3078

**Published:** 2022-12-20

**Authors:** Zainab Suntai

**Affiliations:** Baylor University, Waco, Texas, United States

## Abstract

Research focused on the quality of care received at the end of life has advanced over the past few decades, but few studies have employed an intersectional lens to assess differences in care quality. The theory of intersectionality suggests that individuals with membership in two or more vulnerable groups may be at risk of experiencing increased hardships and stressors across the lifespan. To test the theory of intersectionality and fill the gap in research, this study aimed to assess the combined impact of race and gender on the quality of care received at the end of life among older adults. Data were derived from the combined Round 3 to Round 10 of the National Health and Aging Trends Study, an annual longitudinal panel survey of adults aged 65 and older in the United States. Chi-square tests were used for bivariate analyses and two multivariate logistic regressions were run. Results showed that White men were the most likely to have had excellent or good care at the end of life, followed by White women, Black men, and then Black women. This points to a significant disadvantage for Black women, who lag behind all other groups in other life domains such as income, education, and access to health insurance. Interventions may include intersectionality-focused cultural humility training, cultural matches between patients and providers, and the adoption of a universal health insurance plan to reduce the gaps in service quality caused by the dominance of employment-based health insurance in the United States.

